# Vitamin E ameliorates oral mucositis in gamma-irradiated rats (an in vivo study)

**DOI:** 10.1186/s12903-023-03408-x

**Published:** 2023-09-27

**Authors:** Salwa Farid Ahmed, Mostafa A. Bakr, Amr H. Rasmy

**Affiliations:** Health Radiation Research Dept, National Center for Radiation Research and Technology, Egyptian Atomic Energy Authority, Cairo, Egypt

**Keywords:** Vitamin E, Mucositis score, Radiation-induced oral mucositis, Apoptosis, Proliferation

## Abstract

**Background:**

Radiation therapy is the primary treatment for neck and head cancer patients; however, it causes the development of oral mucositis accompanied by tissue structure destruction and functional alteration. This study was conducted to evaluate the effect of different doses of vitamin E as a treatment for radiationinduced oral mucositis in rat model.

**Methods:**

35 male albino rats were randomly divided into five groups: control, untreated radiation mucositis (single dose of 20 Gy), treated radiation mucositis; radiation (single dose of 20 Gy) then vitamin E at doses of 300, 360 and 500 mg/Kg for seven days started 24 h after irradiation. Body weight and food intake were evaluated for each rat. The mucositis score was assessed every day. Rats were sacrificed once at the end of the experiment, and tongue specimens were stained with hematoxylin and eosin, anti P53 and anti Ki67 antibodies.

**Results:**

Results indicated more food intake and less weight reduction in vitamin E treated groups and the contrary for gamma-irradiated group. Additionally, vitamin E delayed the onset and decreased the severity and duration of mucositis. It also restored the histological structure of lingual tongue papillae. Vitamin E treated groups showed a significant higher Ki67 and lower P53 expression as compared to untreated radiation group. The overall improvement increased as vitamin E dose increased. Finally, the amelioration can be attributed to the decreased apoptosis and increased proliferation of cells.

**Conclusions:**

Vitamin E especially at dose of 500 mg/Kg could be an effective treatment for radiation-induced oral mucositis.

**Supplementary Information:**

The online version contains supplementary material available at 10.1186/s12903-023-03408-x.

## Introduction

Radiotherapy (RT) is a treatment of neck and head cancer patients; however, it may lead to oral mucositis (OM), tissue destruction or functional alteration [1]. Dry mouth and loss of taste are also side effects of radiotherapy that may persist long after the therapy stops [2]. Managing OM based on the pathobiology of the radiation-induced oral mucositis [1]. On molecular base, mucositis starts with the formation of reactive oxygen species (ROS), followed by the release of pro-inflammatory cytokines, as interleukin-1 beta (IL-1 β) and tumor necrosis factor (TNFα), resulting in infection, mucosal damage and cell damage consequently loss of their function [3].

Vitamin E has an important role, which is scavenging free radicals thus preventing radiation-induced damage of cell membrane [4]. Free radicals cause an oxidative stress and lipid peroxidation that may lead to cell death [5].

Vitamin E deficiency due to inadequate dietary intake enhances lipid peroxidation that result in an oxidative stress. Alternatively, high concentration of vitamin E suppresses lipid peroxidation and scavenges ROS generated in metabolism [6]. Physiological concentration of ROS is important for the regulation of intracellular pathways [7], however high concentration is certainly harmful as it causes oxidative stress, cellular dysfunction and the incidence of several diseases [8].

A reduction in the unfavorable effects of radiotherapy will improve the quality of life of patients. Thus, the aim of this study was to evaluate the effect of different doses of vitamin E as a treatment for radiationinduced oral mucositis in rat model.

## Methods

### Ethical considerations & animals

The experiment followed the protocol approved by the Research Ethics Committee of the National Center for Radiation Research and Technology (REC-NCRRT), Egyptian Atomic Energy Authority, no. 36 A/ 22. Thirty-five adult male albino rats, 8–12 weeks old and weighed 220 ± 20 g. at the time of irradiation were included. Rats were housed in polypropylene/stainlesssteel cages of dimensions 54, 37 and 27 cm (5 rats/cage), submitted to suitable ventilation, 55% humidity, 25:28 °C temperature, light/dark regime (12 h of light/dark cycle) with pellet diet and drinkable water ad libitum. The investigators and the workers of the Egyptian Atomic Energy Authority’s animal house were responsible for the care of animals; however, euthanasia was the investigators responsibility. No humane ends were expected during the study.

### Grouping

Rats were randomly divided into five groups (n = 7); group C: negative control (Neither irradiation nor treatment was given), group R: rats were subjected to localized gamma-irradiation only, however for groups R + 300 VE, R + 360 VE and R + 500 VE: rats were subjected to localized gamma-irradiation followed by 300, 360 and 500 mg/Kg/day oral vitamin E for 7 days, respectively [9–11].

### Gamma-irradiation

Before localized irradiation, rats of groups R, R + 300 VE, R + 360 VE and R + 500 VE were anesthetized with an anesthetic combination of ketamine 10% and xylazine 2% (2:1) (0.12 mL/100 g body weight) which was applied to the intraperitoneal region of each rat [12]. Rats were then subjected to a single dose of localized (cranium) gamma-irradiation at dose of 20 Gy using the Indian Gamma Cell (^60^Co) at the National Centre for Radiation Research and Technology (NCRRT), Egyptian Atomic Energy Authority, Cairo, Egypt [13]. They were completely immobilized inside a special shield then exposed to the previously mentioned dose of gamma radiation at dose rate of 11.82 Gy/min.

### Vitamin E treatment

All rats of groups R + 300 VE, R + 360 VE and R + 500 VE received oral vitamin E (Pure vitamin E, Pharco Pharmaceuticals, Egypt) using an oral gavage at doses of 300, 360 and 500 mg/Kg/day respectively for seven days started 24 h after gamma irradiation [14].

### Euthanasia of animals

At the end of the experiment, rats were sacrificed by an over dose anesthesia (ketamine).

### Clinical evaluation

Body weight and the amount of food intake were evaluated for each rat every two days. Additionally, tongues of irradiated rats were daily examined for the clinical signs of mucositis using a mucositis score (score 0; normal, score 0.5; slightly pink, score 1; slightly red, score 2; severe reddening, score 3; focal desquamation, score 4; exudation or crusting covering less than one-half of lip, score 5; exudation or crusting covering more than one-half of lip) [15].

### Histological evaluation

Tongues were dissected and fixed in 10% formalin for 48 h, embedded in paraffin, and cut into 5-μm sections. Sections were then stained with hematoxylin and eosin (H&E) for histological assessment. Other sections were stained with antiP53 and anti-Ki67 for detecting cell death and proliferation. The mean number of P53 and Ki67 expressing cells were calculated in ten different fields (400 X) using Leica Qwin 500 software.

### Statistical analysis

Data were subjected to one way-analysis of variance (ANOVA) and expressed as mean and standard deviation. Multiple range tests were used when differences among groups were significant. Statistical analysis was done using Statgraphics 18 software, Statpoint Technologies, Inc., 560 Broadview Ave. Warrenton, Virginia 20,182. *P value* was set to be ≥ 0.05.

## Results

### Clinical evaluation

#### Body weight

**Fig. 1:**
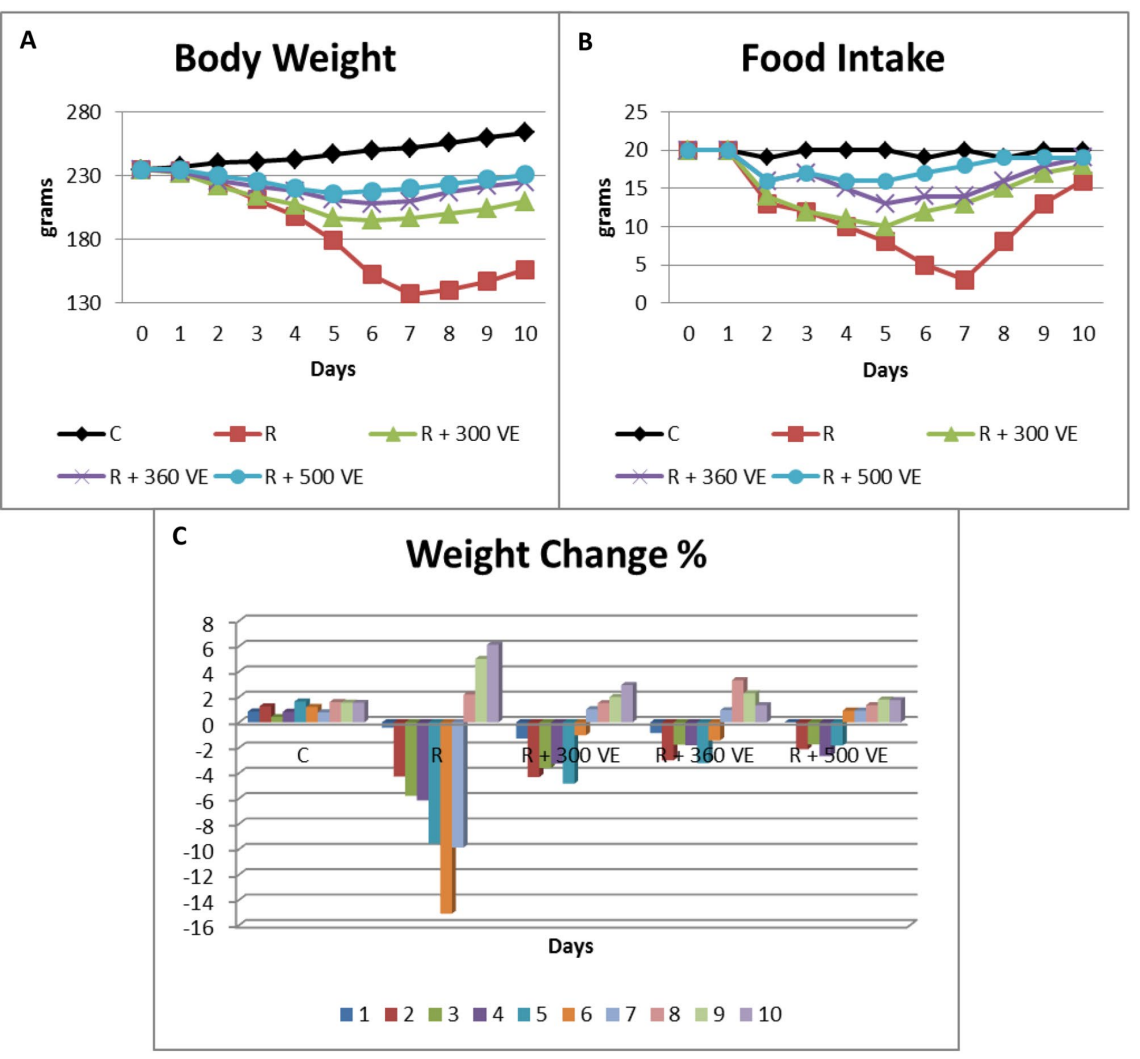
Chart represents (**A**) rats’ body weight (g.), (**B**) food intake (g.) and (**C**) the percentage of weight change of different groups throughout the experimental period

Body weight increased gradually for rats of group C from day one to ten. In contrast, rats of group R showed marked weight reduction from the first to the seventh day followed by weight gain until the tenth day but it did not reach normal (156 g.) (About 150 ± 10 g.). The maximum percentage of weight reduction was 15.1% at day 6. Rats of group R + 300 VE showed noticeable decrease in weight from day one to six followed by slight improvement however; it did not reach normal (210 g.) (About 210 ± 10 g.). The weight of group R + 360 VE rats showed similar response as those of group R + 300 VE with more weight improvement at the end of the experiment. The maximum weight reduction was 4.8% and 3.3% at day 5 for group R + 300 VE and group R + 360 VE, respectively. Rats’ weight of group R + 500 VE exhibited unnoticeable weight reduction from the second to the fifth day then weight started to increase and it almost reached the weight that the experiment started with at the tenth day. Maximum weight reduction (2.7%) was detected at day four (Fig. 1A & C).

#### Food intake

Food intake of rats of group C stayed constant throughout the study period, on the contrary, group R showed marked gradual decrease in rats’ food intake from day 2 to 7 followed by gradual increase but did not reach normal at the end of the experiment. Rats of groups R + 300 VE, R + 360 VE and R + 500 VE showed moderate reduction in food intake till the 5th day, however R + 300 VE was the worst, then started to increase and approximately reached normal at the end of the study, however R + 500 VE reached almost normal from day 8 (Fig. 1B).

#### Mucositis score

In group C, no significance was observed as compared to previous date in the same group throughout the experimental period. In group R, the first sign of mucositis appeared at day one in six of seven rats. There was a gradual significant increase in mucositis score until day six however, no significance was reported between day three & four. Nevertheless, an insignificant decrease was observed between day six & seven. On the other hand, significant decrease in mucositis score from day seven to ten was noticed to reach score of 0.33 that showed insignificance when compared with the control (Table 1).

**Table 1:** Clinical mucositis score (mean ± S.D) among different groups throughout the experimental period

		C	R	R + 300 VE	R + 360 VE	R + 500 VE	
Days	1	0.00 ± 0.000 a	0.00 ± 0.000 a	0.00 ± 0.000 a	0.00 ± 0.000 a	0.00 ± 0.000 a	*p*-value 0.000
	2	0.00 ± 0.000 a	0.50 ± 0.316 bcd	0.33 ± 0.258 abcd	0.25 ± 0.274 abc	0.17 ± 0.258 ab	
	3	0.00 ± 0.000 a	1.08 ± 0.492 fg	0.75 ± 0.274 def	0.67 ± 0.258 cdef	0.58 ± 0.376 bcde	
	4	0.00 ± 0.000 a	1.67 ± 0.516 ghi	1.50 ± 0.548 gh	1.25 ± 0.612 g	1.00 ± 0.548 efg	
	5	0.00 ± 0.000 a	2.67 ± 0.516 k	2.50 ± 0.548 k	2.00 ± 0.632 ij	1.42 ± 0.665 gh	
	6	0.00 ± 0.000 a	3.83 ± 0.753 l	1.83 ± 0.408 hi	1.33 ± 0.753 g	0.83 ± 0.258 defg	
	7	0.00 ± 0.000 a	3.50 ± 0.548 l	1.00 ± 0.548 efg	0.67 ± 0.258 cdef	0.58 ± 0.204 bcde	
	8	0.00 ± 0.000 a	2.33 ± 0.816 jk	0.58 ± 0.204 bcde	0.17 ± 0.258 ab	0.08 ± 0.204 a	
	9	0.00 ± 0.000 a	1.33 ± 0.516 g	0.33 ± 0.258 abcd	0.00 ± 0.000 a	0.00 ± 0.000 a	
	10	0.00 ± 0.000 a	0.33 ± 0.258 abcd	0.00 ± 0.000 a	0.00 ± 0.000 a	0.00 ± 0.000 a	

Different letters mean significant difference at *P* ≤ 0.05

In group R + 300 VE, mucositis appeared at day one in four of seven rats. The mucositis score showed gradual increase with insignificant difference until day three that became significant until day six. Then, the mucositis score decreased significantly at day six and seven followed by insignificant reduction until day ten. The mucositis disappeared completely at day ten and lastly mucositis score reached normal (score 0). In group R + 360 VE, three of seven rats developed first mucositis sign at day one. A gradual insignificant increase from day one to three followed by significant increase from day four to five in mucositis score. Yet, a gradual significant decrease from day six to eight was noticed. At day nine & ten, mucositis score decreased to reach normal score. In group R + 500 VE, only two rats of seven exhibited first mucositis sign at day one. Mucositis score exhibited an insignificant increase till day five comparing each day with the previous one, followed by an insignificant decrease at day six & seven and finally reached normal score at day eight, nine and ten (Table 1).

At day one, no significant difference was observed between all groups. At day two, no significant difference was observed between treatments groups score and either C or R group, however R group showed a significantly higher mucositis score as compared to group C. At day three, all groups exhibited significantly higher mucositis score than that of group C. The highest mucositis score was detected in group R that was significant only when compared to group R + 500 VE. At day four, significance was revealed between group C and all other groups. Moreover, there was no significant difference between R group and other treatments groups. At day five, significance was recognized between group C and all other groups. Both, R and R + 300 VE groups showed significantly higher score compared to R + 360 VE and R + 500 VE groups, which both in turn showed significant, difference between each other (Table 1).

At days six and seven, all groups had a significant higher mucositis score as compared to group C. However, all treatments groups showed mucositis score significantly smaller when compared to group R with insignificant between them. At day eight, only R and R + 300 VE groups had significantly higher mucositis score as compared to group C. In addition, significance was noted between group R and all other treatments groups with no significant difference between them except between group R + 300 VE and group R + 500 VE. At day nine, insignificance was monitored between group C and all other groups except group R that by its turn showed a significant increment with all other groups. At day ten, once more as in day 1, no significance was manifested between all groups (Table 1). Analysis of Table (1) data revealed that the normal score (0) was reached by the different used treatments at day nine for group R + 360 VE and group R + 500 VE while for group R + 300 VE at day ten.

### Histological evaluation

#### Hematoxylin & eosin

**Fig. 2:**
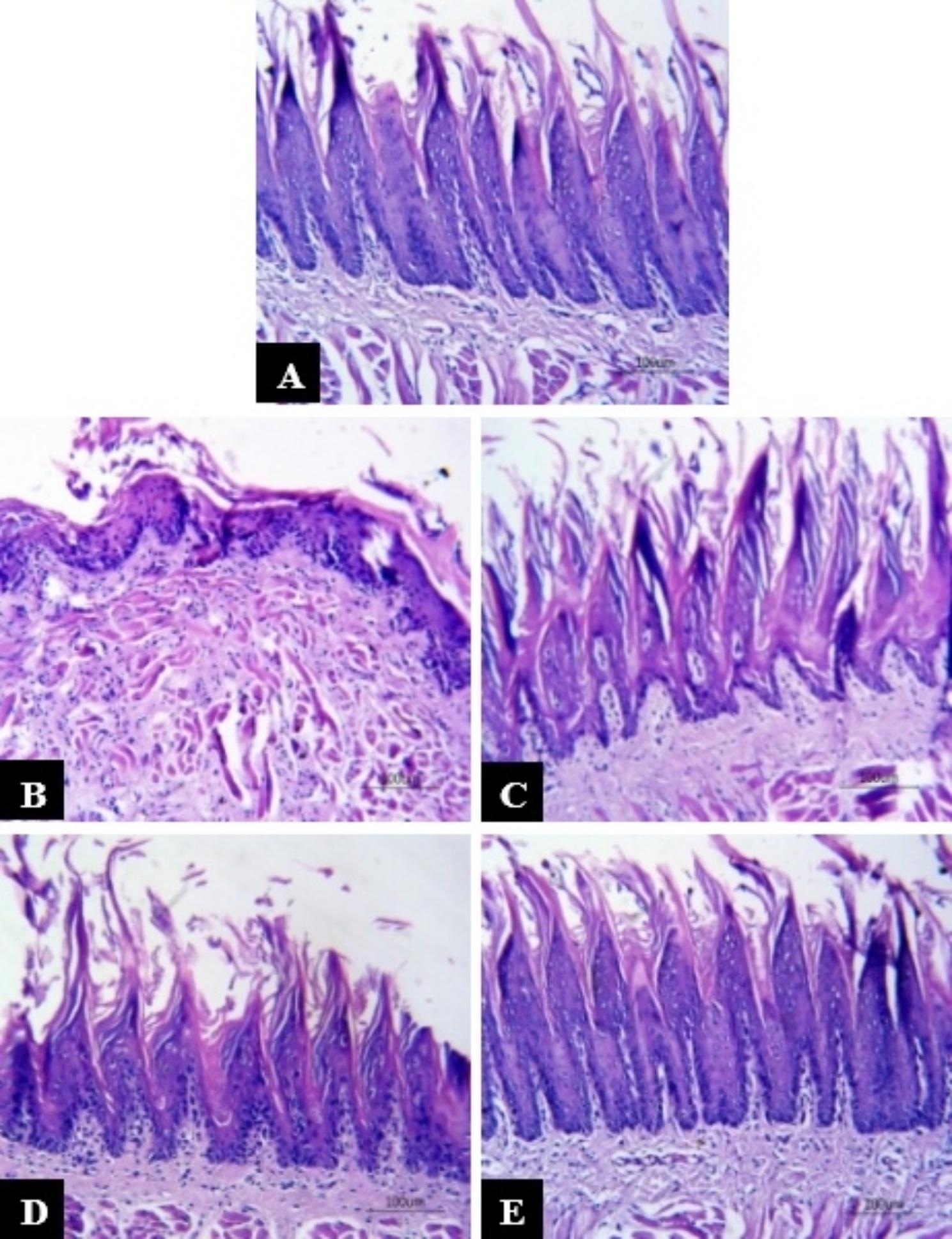
Photomicrograph of the tongue dorsal surface (**A**) group C showing stratified squamous epithelium, elongated filiform papillae with thin keratin, intact basement membrane and no inflammatory infiltrate in the submucosa (**B**) untreated mucositis R group revealed complete loss of lingual papillae, disrupted epithelium, area of absent keratin layer and irregular basement membrane, (**C**) mucositis R + 300 VE group showed short lingual papillae, vacuolated epithelial cells and intact epithelium with irregular keratin layer, (**D**) mucositis R + 360 VE group revealed lingual papillae with more preserved structure, intact basement membrane and more uniform keratin layer, (**E**) mucositis R + 500 VE group exhibited long, conical shaped lingual papillae of normal structure with slight cytoplasmic vacuolization, intact basement membrane and uniform keratin layer [H & E]

Tongue of group C composed of stratified squamous epithelium covering the dorsal surface. The filiform papillae were elongated, conical shaped and covered with thin uniform keratin layer. The basement membrane was intact forming well-defined secondary connective tissue. The submucosa composed of fibroblasts, many blood vessels and well-arranged collagen fibers with no inflammatory infiltrate (Fig. 2A). Examined tongue specimens of mucositis group R revealed stratified squamous epithelium covering the dorsal surface with complete loss of papillae and partial loss of the keratin layer. The thickness of the epithelium decreased with irregular basement membrane. The basal cells exhibited disturbed alignment. The underlying submucosa showed decreased thickness with areas of complete loss and infiltrated with inflammatory cells (Fig. 2B). The tongue specimen of R + 300 VE showed stratified squamous epithelium covering the dorsal surface partially preserved, short lingual papillae. Some epithelial cells revealed cytoplasmic vacuolization. The lingual papillae had well defined secondary connective tissue with irregularly distributed basal cells. The keratin layer showed different thickness. The submucosa showed decreased fibroblast with little inflammatory cells (Fig. 2C). In group R + 360 VE, the dorsal tongue surface was covered with stratified squamous epithelium with more preserved lingual papillae. The length of the lingual papillae was higher than those of R and R + 300 VE groups. The basal cells showed irregular alignment. The keratin layer was continuous with different thickness. The submucosa presented increased fibroblast number with no inflammatory cells (Fig. 2D). Regarding R + 500 VE group, the dorsal tongue surface covered with stratified squamous epithelium with almost normal lingual papillae. The normal height and alignment were restored. Some epithelial cells still exhibited cytoplasmic vacuolization. The basal cells showed alignment that is more regular. The epithelium covered with uniform thin keratin layer. The underlying submucosa contained fibroblast, blood vessels and well-formed collagen fibers without inflammatory infiltrate (Fig. 2E).

#### Anti-P53 immunohistochemistry

**Fig. 3:**
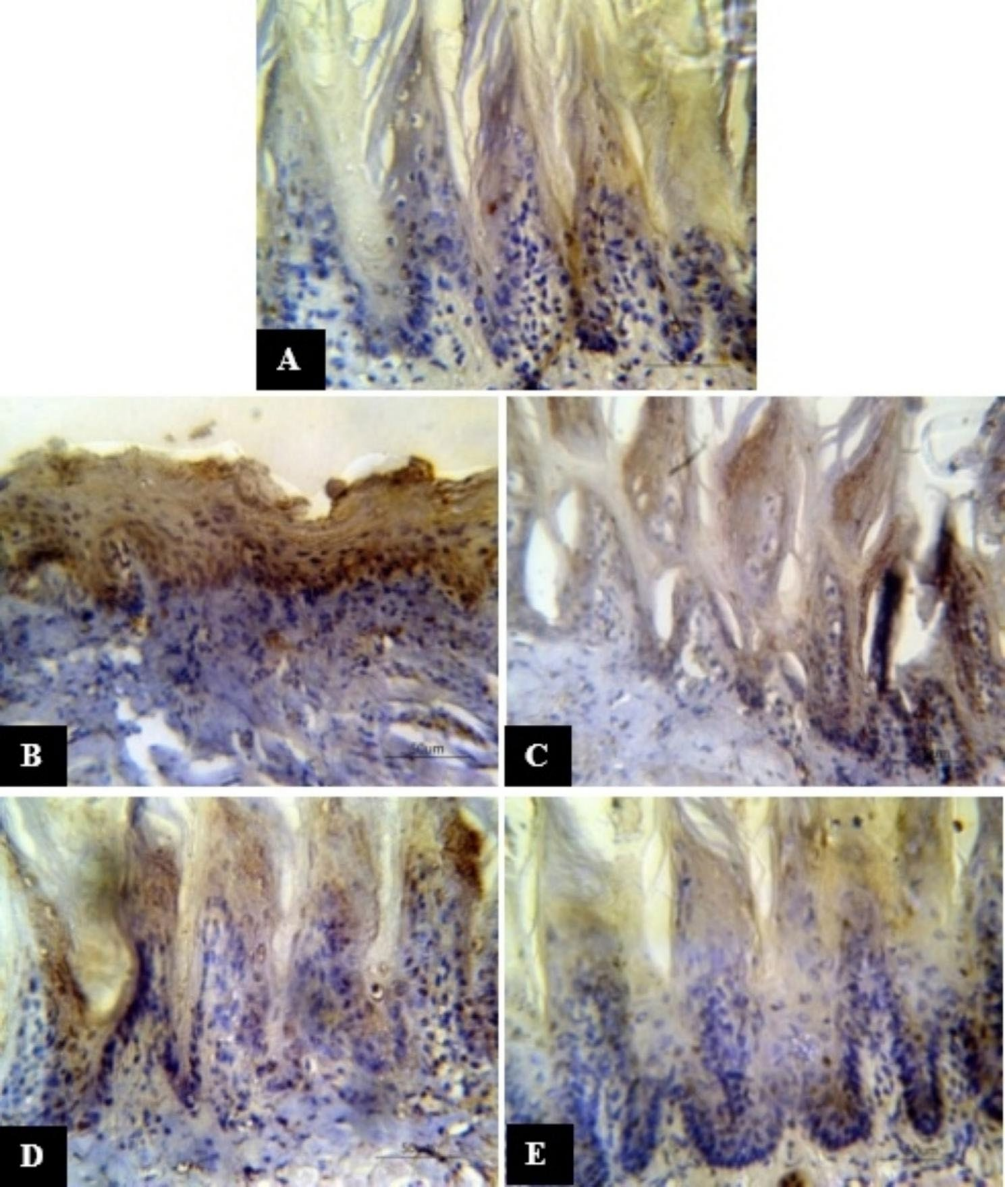
Photomicrograph of P53 expression in the tongue dorsal surface of (**A**) group C with few positive cells, (**B**) untreated mucositis R group with numerous positive cells, (**C**) mucositis R + 300 VE group with many positive cells, (**D**) mucositis R + 360 VE group with some positive cells, (**E**) mucositis R + 500 VE group with few positive cells

The tongue dorsal surface of C group revealed few positive P53 expression cells. For untreated mucositis R group, numerous cells along the epithelium exhibited positive P53 expression. Group R + 300 VE showed many P53 positive cells involving the whole epithelium thickness. In group R + 360 VE, some papillae contained many positive cells while others contained only few positive cells. On the other hand, the dorsal tongue surface of group R + 500 VE revealed only few positive cells in some papillae (Fig. 3A, B, C, D and E respectively).

Data concerning the mean number of P53 expressing cells of all groups was presented in Table (2). The mean number of P53 expressing cells was higher in all groups as compared to the control however, the difference was significant with groups R, R + 300 VE and R + 360 VE (*p* ≤ 0.01) with no significance with group R + 500 VE. As compared to group R, all treatments groups exhibited significantly lower values of mean number of P53 expressing cells. As the dose of VE increased, the mean number of P53 expressing cells significantly decreased.

**Table 2:** Mean number of P53 and Ki67 expressing cells of different experimental groups

	Mean No of P53 expressing cells	Mean No of Ki67 expressing cells
C	6.3 ± 1.16 a	48.8 ± 1.03 a
R	54.7 ± 6.41 b	13.7 ± 2.36 b
R + 300 VE	36.8 ± 4.98 c	31.8 ± 2.49 c
R + 360 VE	27.9 ± 4.48 d	38.1 ± 1.97 d
R + 500 VE	9.1 ± 1.60 a	47.0 ± 2.05 a
*p*-value	0.000	0.000

Different letters mean significant difference at *P* ≤ 0.05

#### Anti-Ki67 immunohistochemistry

**Fig. 4:**
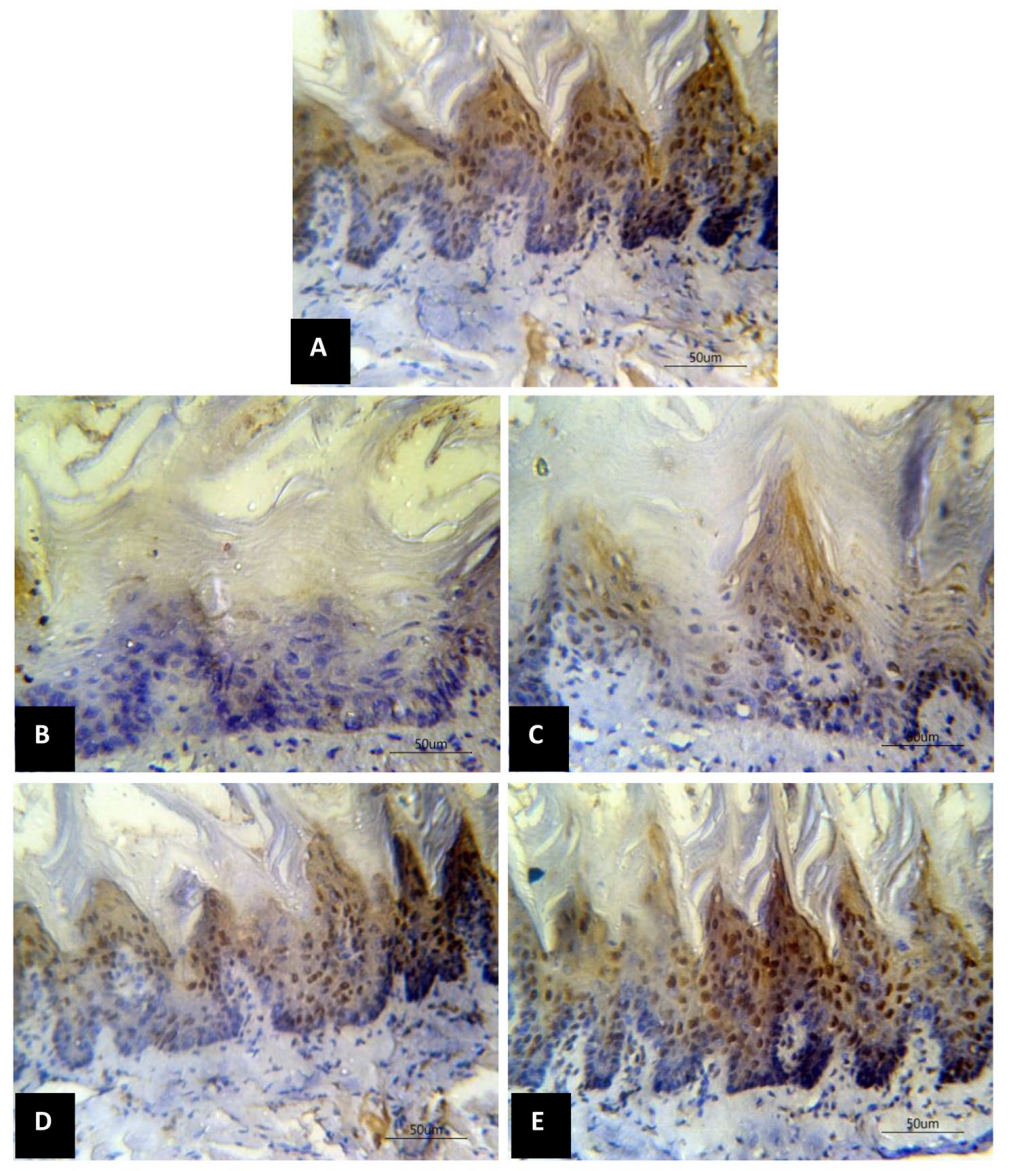
Photomicrograph of Ki67 expression in the tongue dorsal surface (**A**) group C showed numerous positive cells, (**B**) untreated mucositis R group showed few positive cells, (**C**) mucositis R + 300 VE group with some cells showed positive expression, (**D**) mucositis R + 360 VE group with some basal and numerous supra basal cells positively expressed Ki67, (**E**) mucositis R + 500 VE group with numerous basal and supra basal cells expressing Ki67

The expression of Ki67 was evident in numerous cells of group C tongues mostly in suprabasal cells. Untreated mucositis group R showed only few positive cells. Group R + 300 VE presented few basal and many suprabasal positive cells. Many basal and numerous suprabasal positive Ki67 cells were detected in R + 360 VE group. The dorsal surface of group R + 500 VE exhibited numerous Ki67 positives cells involving whole the epithelial thickness (Fig. 4A, B, C, D and E, respectively).

Comparing the mean number of Ki67 expressing cells among groups, all groups revealed a statistically significant reduction as compared to group C except group R + 500 VE where the reduction was insignificant. Treatments groups had a significantly higher mean number of Ki67 expressing cells when compared to group R. Moreover, the increment in the mean number of Ki67 expressing cells significantly increased as the dose of VE increased (Table 2).

## Discussion

RT of the oral cavity or neck can be extremely harmful. RT can cause erythematous, erosive and ulcerative OM in about one fifth of the patients [16]. OM induced by radiotherapy for neck and head cancers reduces the quality of life and has various symptoms; pain, burning sensation and inflammation of the mucosal lining and is associated with dehydration and weight loss. Consequently, drinking, eating, swallowing and even talking can become very difficult. Hence, OM needs to be controlled specially in high-risk patients as radiotherapeutic patients [17]. Yet, effective therapeutic agents are lacking and only symptomatic treatments as local anesthesia, analgesics, antiulcer& anti-inflammatory agents and low-level laser irradiation are available. However, the clinical response obtained by these drugs against OM is poor [16].

Vitamin E is the most important natural non-enzymatic lipid-soluble antioxidant in the animal body. It has a potent antioxidant effect on tissues through its scavenging activity of ROS (superoxide and hydroxyl radicals) that modulate the signal transduction pathways [18]. Additionally, it protects cell membranes and lipoproteins against lipid peroxidation because; it stabilizes cell membrane by influencing cell signaling and it breaks the lipid radical chain reaction [6]. It also has radio-protective effects such as DNA repair and reduction in the number of micronuclei of lymphocytes as well as the adverse effects of radiotherapy [19].

The protective effect of vitamin E against radiation damage is still debatable. Ferreira et al. [20] concluded that 400 IU vitamin E minimizes the prevalence of radiation-induced OM. Moreover, the use of 40 mg/kg/day vitamin E has been proved to delay the onset and severity of OM [21]. On the other hand, Gomes et al. [9], declared that gamma irradiation caused acinar tissue damage in gamma-irradiated rats; nevertheless, vitamin E had no effect as a radio-protective agent on the parotid glands of them. Thus, the aim of our study was to examine the effect of different doses of vitamin E as a treatment for radiationinduced oral mucositis in rat model. The effect of topical and systemic vitamin E against oral mucositis in chemotherapeutic children was examined and results proved that topically applied was more successful [22].

Rats of group R showed marked weight and food intake reduction followed by weight gain and increased food intake that did not reach normal until the end of the study. On the other hand, groups R + 300 VE, R + 360 VE and R + 500 VE revealed variable moderate degrees of weight loss and food intake reduction for a short period then both increased with preference to the higher VE dose. These results match that of Nakajima et al. [16] who stated that the body weight and the intake of food decreased after subjecting mice to 20 Gy x-ray irradiation. Moreover, Üçüncü et al. [21] and Cihan and Deniz [23] elucidated that a noticeable reduction in gamma-irradiated rats’ weight and food intake compared to normal was noted. Additionally, Azman et al. [24] confirmed that rats that were fed on 30 mg/kg vitamin E showed steady improvement in body weight throughout the study period. In contrast, dietary vitamin E concentration 25, 250 or 2500 mg/kg had no effect on either body weight or the intake of food of rats as demonstrated by Eder et al. [25]. Dissimilar findings may be attributed to the difference in vitamin E dose or the variation in the frequency of recording food intake.

In both groups R and R + 300 VE, there was a gradual increase in mucositis score till day 6 followed by decrease from day 7 to 10 however mucositis disappeared at day 10 only in group R + 300 VE. In accordance with our results was that of Cihan and Deniz [23] who stated that gamma irradiation caused significant increases in the severity of OM. In both groups R + 360 VE and R + 500 VE gradual increase in mucositis score occurred till day 5 then gradual reduction that reached normal at day 9 and day 8, respectively. Üçüncü et al. [21] clarified that the severity of mucositis score was significantly higher in rats subjected to gamma irradiation than those treated with vitamin E after being subjected to gamma irradiation. Otherwise, no sign of OM was observed in the mucosa of gamma-irradiated rats nor did they lose weight, however the weight gain rate was inferior in irradiated rats as stated by de Moraes et al. [19], yet using different radiation dose may explain the diverse results.

The untreated mucositis group revealed loss of lingual papillae, basement membrane irregularity with inflammatory cells detected in the underlying submucosa. Groups treated with different doses of vitamin E provided partial to almost complete restoration of the lingual papillae structure and height. Vitamin E treatment also decreased the inflammatory infiltrate in the underlying submucosa with absence of inflammation with the high vitamin E dose (500 mg/Kg). Similar result was obtained by Nor-Eldin and Elsayed [11] who found that 300 mg/Kg vitamin E provided a protection for brain and cerebellum against whole body irradiation at eight Gy. Similarly, Saleh et al. [26] found that vitamin E (40 mg/Kg/day) reduced the cytotoxic effect of cyclophosphamide with regard to rats’ tongues. On day four, the dorsal surfaces of tongues showed a significant increase in epithelial thickness and keratin layer as compared to cyclophosphamide group accompanied with partial restoration of filiform and fungiform papillae morphology. Congestion and inflammatory infiltrate were also detected in the connective tissue. Four days later, acanthosis of the epithelium was still existed with almost normal fungiform papillae and their taste buds. Epithelial vacuolization were revealed but less than those of cyclophosphamide group. Moreover, vitamin E treatment significantly reduced damage score as compared to cyclophosphamide group along the experiment.

On contrary, 360 mg/kg vitamin E gave only partial protection of the parotid glands against 15 Gy localized irradiation [9]. Many antioxidants other than vitamin E displayed a successful protection against radiationinduced mucositis. Alpha lipoic acid at dose of 100 mg/kg improved the oral mucosa injury due to exposure to a single 18 Gy localized irradiation [27]. Pretreatment with N-Acetylcysteine, another antioxidant, was able to prevent radiation injury of buccal mucosa through inhibition of intracellular reactive oxygen species production in addition to prevention of autophagy and inflammation of buccal mucosa [28]. The damage of tongue tissue was mainly due to reactive oxygen species (ROS) generated by radiation that led to oxidative stress and lipid peroxidation and consequently cell death [5]. The histological improvement of tongue tissue due to vitamin E treatment could be attributed to its antioxidant capacity inhibiting ROS induced cell membrane injury as well as being a membrane stabilizer agent facing the damage occurred by phospholipids [29].

P53 is a tumor suppressor and a multifunctional transcription factor that is considered the prime controller regarding the response of cells to radiation [30]. The level of P53 protein rises in cells due to the enhancement of protein translation and the inhibition of protein degradation as a result of DNA damage occurred after radiation exposure [31]. Accumulation of p53 protein in the nucleus provokes cell cycle arrest and facilitates DNA repair that promotes cell survival or conversely, stimulates apoptosis and senescence of cells that augments cell death [32]. P53 can skip being destructed and accumulates thus can be detected by immunestaining. Mutation frequency of P53 is thought to be about 50% in human cancers [30]. We selected the P53 protein in the study because it is a good marker of dysplasia sites.

Our results proved that P53 expressing cells was higher in all groups as compared to the control except group R + 500 VE where no significance was detected compared to normal, whereas group R was significantly higher than all other groups. Additionally, P53 expressing cells, significantly decreased as the dose of VE increased. Our results were in accordance Zhang et al. [33] who reported a significant increase in P21 expression in mice tongue exposed to single or fractionated radiation. In a previous study, no clear epithelial dysplastic changes were displayed in the specimens of rats’ tongues, however a minimal non-significant raise in the P53 expression in the diabetic rats group was found [34]. In accordance with our finding, Yeoh et al. [35] found that P53 expression extraordinarily increased in irradiated rats specimens other than untreated controls. Besides, El-Rouby and El-Batouti [36] showed that P53 considerably reduced in rats treated with 40 mg/kg vitamin E for five consecutive days. Inconsistent with our results, P53 increased significantly in rats that were given vitamin E compared to other groups as revealed by Dashtiyan et al. [37]; however, this may be due to giving vitamin E for 6 weeks or due to high lipid levels as the authors demonstrated. In addition, it was shown that vitamin E treatment (40 mg/Kg/day) of cyclophosphamide intoxicated rats significantly reduced the mean caspase-3 immune expressions of tongues epithelium at day four and eight in comparison to cyclophosphamide group [26].

Ki67 is a nuclear protein that dominates cell proliferation. Consequently, it is considered as a proliferation index of tumors. Several studies indicated that Ki67 could be a significant prognostic factor in oral cancers [38]. We selected the Ki67 is a good marker of cell proliferation. Significant reduction in the mean number of Ki67 expressing cells was observed by comparing group C with all other groups except group R + 500 VE where the reduction was insignificant. Moreover, the increment in the mean number of Ki67 significantly increased as the dose of VE increased. Furthermore, treatments groups had a significantly higher mean number of Ki67 expressing cells compared to group R. Similar result was obtained by Zhang et al. [33] who found a significant reduction in Ki67 and PCNA expression in mice tongue exposed to single or fractionated radiation.

Immunohistochemical detection of PCNA expression in proliferating liver specimens taken from rats that were given a daily oral dose of vitamin E at dose of 500 mg/Kg, showed a positive PCNA immunostaining as verified by Halawani et al. [10] which supports our finding. Cohn et al. [39] similarly emphasized that Ki67 severely reduced in the foliate and circumvallate epithelium after lipopolysaccharide as it stimulates the expression of inflammatory cytokines in taste tissues. Conflicting with us, Toptaş et al. [40] revealed a high rate of Ki67 positive expression in smoker examined individuals. Additionally, It was found that rats intoxicated with cyclophosphamide and treated with vitamin E (40 mg/Kg/day) displayed an increased mean proliferating cell nuclear antigen immune expression in the tongue epithelium at day four and eight compared to cyclophosphamide group [26].

## Conclusion

Vitamin E treatment reduced body weight loss and improved food intake of gamma-irradiated rats. Additionally, it improved oral mucositis and accelerated the restoration of tongue structures. As vitamin E dose increased, the amelioration increased. The amelioration could be due to cell death and proliferation modulation through down regulation of P53 and up regulation of Ki67. Vitamin E especially at dose of 500 mg/Kg could be an effective treatment for radiation-induced oral mucositis.

### Electronic supplementary material

Below is the link to the electronic supplementary material.

Supplementary Material 1

## Data Availability

All data generated or analyzed during this study are included in this published article.
