# Supplementary material for: Vitamin E ameliorates oral mucositis in gamma-irradiated rats (an in vivo study)
Source: BMC Oral Health. 2023 Sep 27;23:697. doi: 10.1186/s12903-023-03408-x (PMC10537122; doi:10.1186/s12903-023-03408-x)

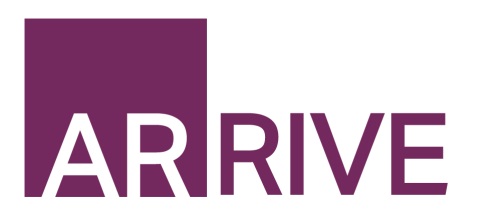


The ARRIVE Guidelines Checklist

Animal Research: Reporting In Vivo Experiments

Carol Kilkenny^1^, William J Browne^2^, Innes C Cuthill^3^, Michael Emerson^4^ and Douglas G Altman^5^

*^1^The National Centre for the Replacement, Refinement and Reduction of Animals in Research, London, UK, ^2^School of Veterinary Science, University of Bristol, Bristol, UK, ^3^School of Biological Sciences, University of Bristol, Bristol, UK, ^4^National Heart and Lung Institute, Imperial College London, UK, ^5^Centre for Statistics in Medicine, University of Oxford, Oxford, UK.*

|  | | ITEM | RECOMMENDATION | Section/ Paragraph |
| --- | --- | --- | --- | --- |
| 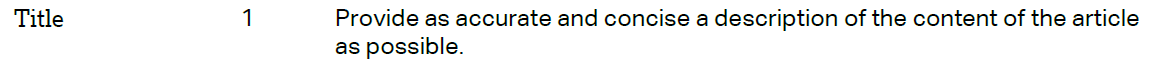 | | | Title |  |
| 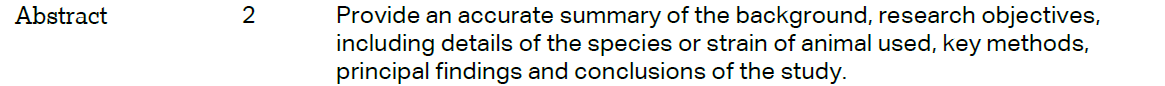 | | | Abstract |  |
| INTRODUCTION | | |  |  |
| 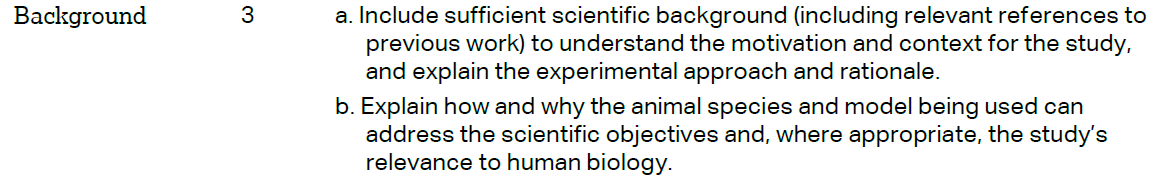 | | | 1, 2& 3 |  |
| 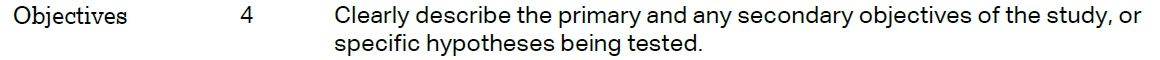 | | | 4 |  |
| METHODS | | |  |  |
| 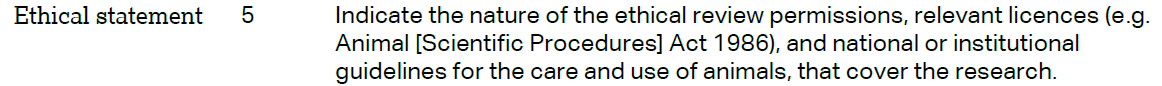 | | | 1 |  |
| 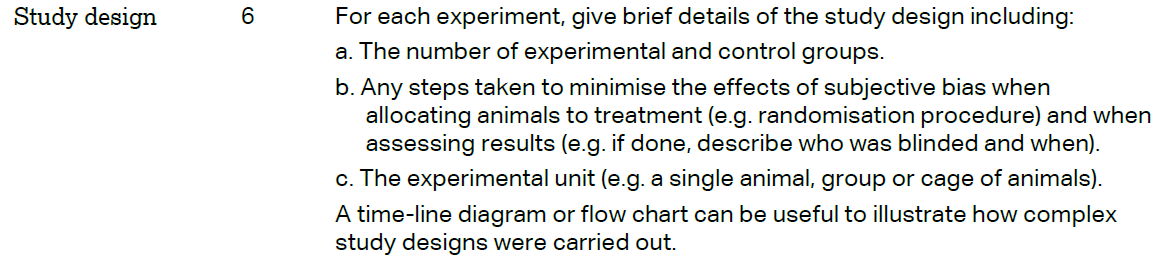 | | | 2 |  |
| 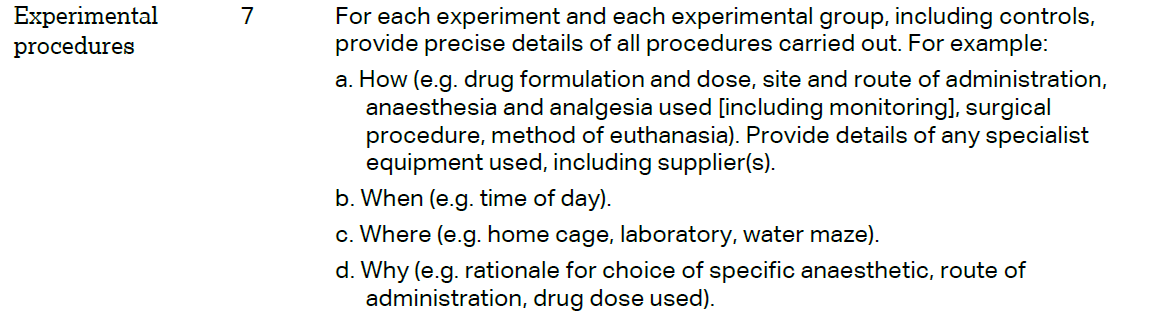 | | | 3, 4& 5 |  |
| 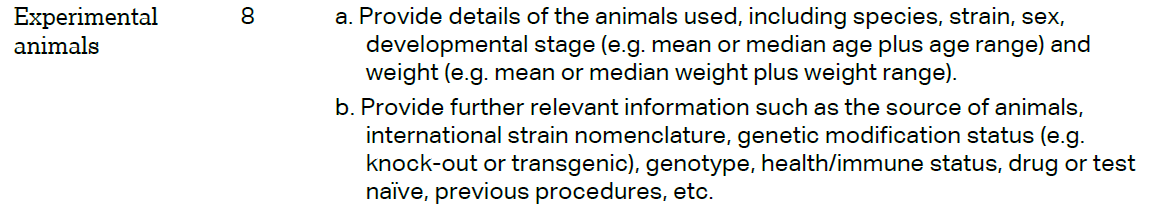 | | | 1 |  |

The ARRIVE guidelines. Originally published in *PLoS Biology*, June 2010^1^

| 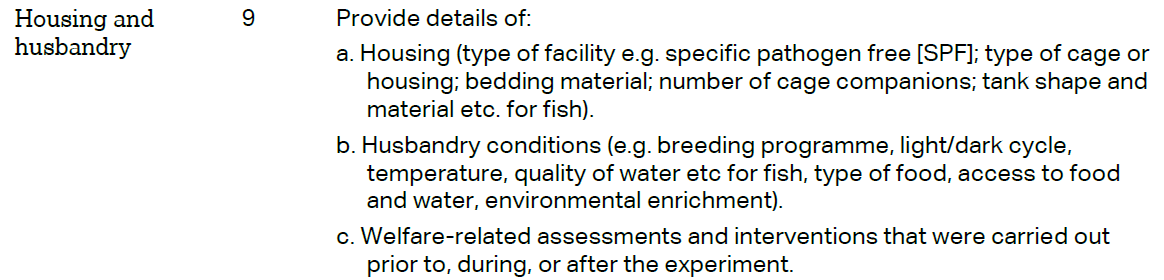 | 1 | |
| --- | --- | --- |
| 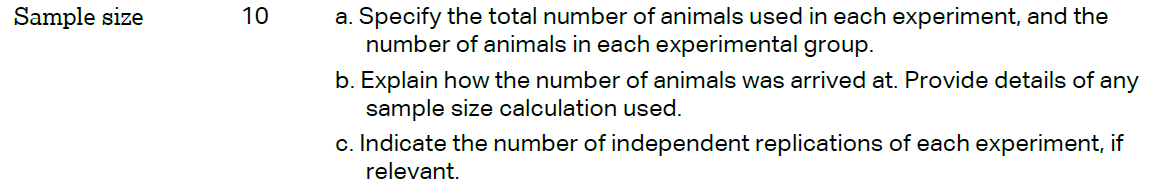 | 1& 2 | |
| 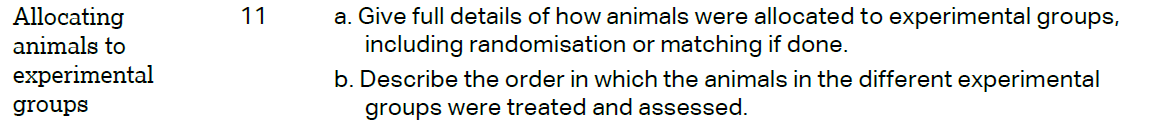 | 2 | |
| 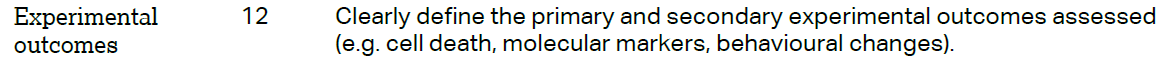 | 6& 7 | |
| 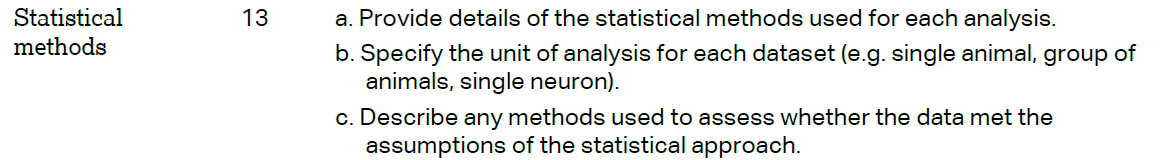 | 8 | |
| RESULTS |  | |
| 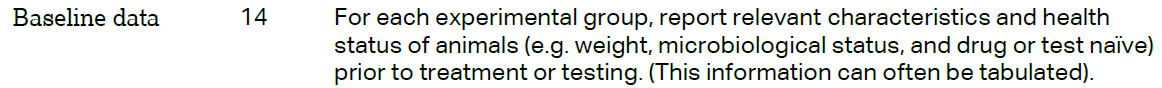 | 1& 2 | |
| 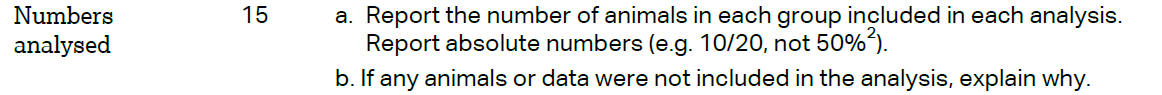 |  | |
| 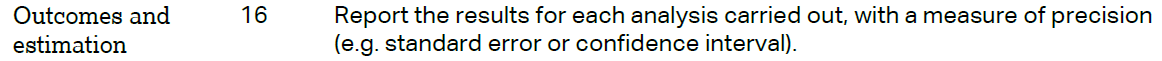 | Results | |
| 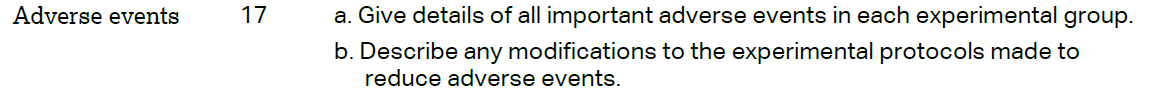 |  | |
| DISCUSSION |  | |
| 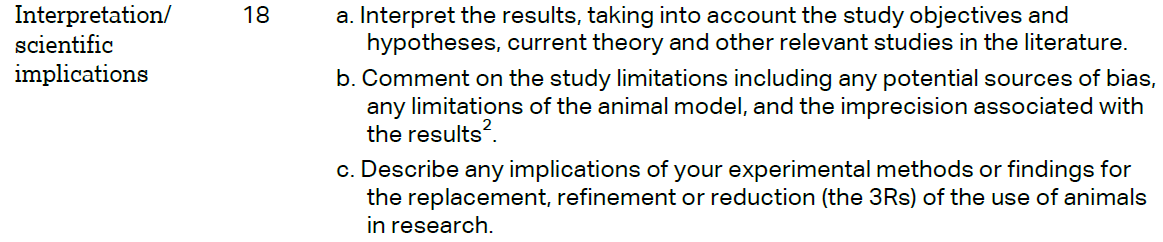 | Discussion | |
| 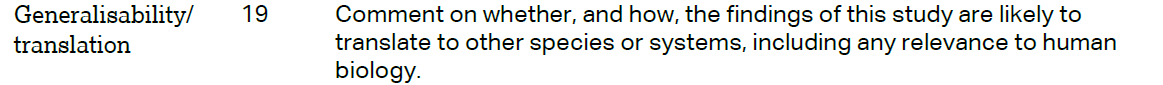 |  | |
| 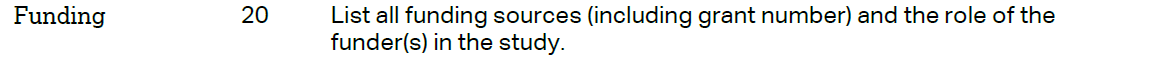 | | No fund |


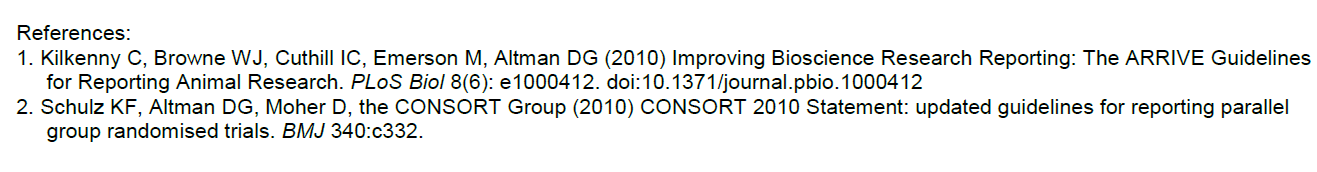

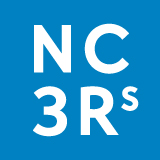

Supplement: Supplementary file 1 — Supplementary Material 1 [file 12903_2023_3408_MOESM1_ESM.docx]
